# Crystal structure of (*E*)-1-(4-meth­oxy­phen­yl)ethanone *O*-de­hydro­abietyloxime

**DOI:** 10.1107/S1600536814016882

**Published:** 2014-08-01

**Authors:** Xiao-Ping Rao, Yan-Jie Cui, Jian-Qiang Zheng

**Affiliations:** a Institute of Chemical Industry of Forest Products, Chinese Academy of Forestry, Key Lab. of Biomass Energy and Material, Jiangsu Province, National Engineering Lab. for Biomass Chemical Utilization, Key and Lab. on Forest Chemical Engineering, SFA, Nanjing 210042, People’s Republic of China

**Keywords:** crystal structure, oxime, de­hydro­abietic acid derivative, biological compounds

## Abstract

In the title compound, C_29_H_37_NO_3_ {systematic name: (*E*)-1-(4-meth­oxy­phen­yl)ethanone *O*-[(1*R*,4a*S*,10a*R*)-7-isopropyl-1,4a-dimethyl-1,2,3,4,4a,9,10,10a-octa­hydro­phenanthrene-1-carbon­yl]oxime}, a new derivative of de­hydro­abietic acid, the two cyclo­hexane rings exhibit a *trans*-ring junction and are in chair and half-chair conformations. The C=N double bond exhibits an *E* conformation.

## Related literature   

For the biological activity of related compounds, see: Cui *et al.* (2013 ▶); Li *et al.* (2008 ▶); Rao *et al.* (2008 ▶); Sepulveda *et al.* (2005 ▶); For the crystal structures of a related compound, see: Rao *et al.* (2009 ▶).
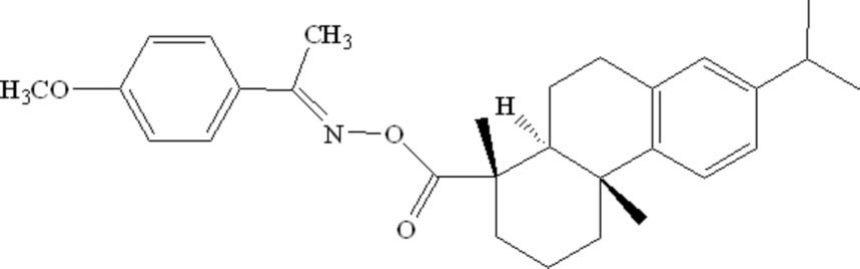


## Experimental   

### Crystal data  

C_29_H_37_NO_3_
*M* *_r_* = 447.60
Orthorhombic, 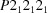

*a* = 6.1700 (12) Å
*b* = 11.051 (2) Å
*c* = 37.526 (8) Å
*V* = 2558.7 (9) Å^3^
*Z* = 4
Mo *K*α radiation
μ = 0.07 mm^−1^
*T* = 293 K
0.30 × 0.20 × 0.10 mm

### Data collection  

Enraf–Nonius CAD-4 diffractometer
Absorption correction: ψ scan (*CAD-4 Software*; North *et al.*, 1968 ▶) *T*_min_ = 0.978, *T*_max_ = 0.993
5399 measured reflections
4691 independent reflections
2211 reflections with *I* > 2σ(*I*)
*R*_int_ = 0.088
3 standard reflections every 200 reflections intensity decay: 1%

### Refinement  

*R*[*F* ^2^ > 2σ(*F* ^2^)] = 0.078
*wR*(*F* ^2^) = 0.183
*S* = 1.00
4691 reflections
298 parameters
1 restraint
H-atom parameters constrained
Δρ_max_ = 0.16 e Å^−3^
Δρ_min_ = −0.17 e Å^−3^

### 

Data collection: *CAD-4 Software* (Enraf–Nonius, 1989 ▶); cell refinement: *CAD-4 Software*; data reduction: *XCAD4* (Harms & Wocadlo, 1995 ▶); program(s) used to solve structure: *SHELXS97* (Sheldrick, 2008 ▶); program(s) used to refine structure: *SHELXL97* (Sheldrick, 2008 ▶); molecular graphics: *SHELXTL* (Sheldrick, 2008 ▶); software used to prepare material for publication: *SHELXTL*.

## Supplementary Material

Crystal structure: contains datablock(s) I, global. DOI: 10.1107/S1600536814016882/lr2129sup1.cif

Structure factors: contains datablock(s) I. DOI: 10.1107/S1600536814016882/lr2129Isup2.hkl

**Fig. 1.**
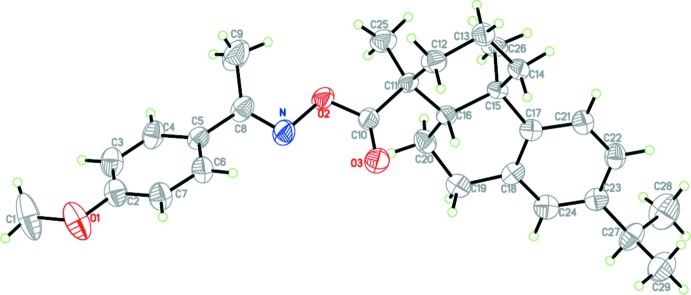
The molecular structure of the title compound, hydrogen atoms are represented by small spheres of arbitrary radius and the displacement ellipsoids are at the 30% probability level.

. DOI: 10.1107/S1600536814016882/lr2129fig1.tif
The mol­ecular structure of the title compound, hydrogen atoms are represented by small spheres of arbitrary radius and the displacement ellipsoids are at the 30% probability level.

CCDC reference: 1015316

Additional supporting information:  crystallographic information; 3D view; checkCIF report

## supplementary crystallographic information

### S2. Structural commentary

De­hydro­abietic acid is an important material for design and synthesis of biological compounds (Li *et al.*, 2008; Rao *et al.*, 2008; Sepulveda *et al.*, 2005). As part of our ongoing project of de­hydro­abietic acid derivatives (Cui *et al.*, 2013, Rao *et al.*, 2009). we report herein the structure of the title compound. The structure of de­hydro­abietyl moiety in the title compound is comparable to that found for de­hydro­abietic acid and related compounds (Rao *et al.*, 2009). There are three six-membered rings, which form planar, half-chair and chair conformations, respectively. the two cyclo­hexane rings are in *trans* ring junction with classic chair and half-chair conformations, respectively, Three chiral centers in the structure exhibit R–, S– and R– configurations, respectively. The C=N double bond is in E configuration.

### S5. Synthesis and crystallization

60 mmol of De­hydro­abietyl chloride in 15 ml CH_2_Cl_2_ were added dropwise to a 60 mmol (4-meth­oxy­phenyl)­ethanone oxime and 60 mmol tri­ethyl­amine dissolved in 40 ml CH_2_Cl_2_ at a temperature 0–5°C. The reaction mixture was allowed to stand at room temperature for 2 h and then washed with water and dried over anhydrous MgSO_4_. The residue was purified by silica gel chromatography.

### S6. Refinement

Crystal data, data collection and structure refinement details are summarized in Table 1. H atoms were positioned geometrically and refined as riding atoms, with C—H = 0.96 Å and *U*_iso_(H) = 1.5*U*_eq_(C) for methyl H atoms, and C—H = 0.97–0.98 Å and *U*_iso_(H) = 1.2*U*_eq_(C,*N*,H) for all other H atoms. Methyl groups were refined in orientation AFIX 137 of program *SHELXL97*.

### Crystal data

**Table:**

C_29_H_37_NO_3_	*F*(000) = 968
*M**_r_* = 447.60	*D*_x_ = 1.162 Mg m^−^^3^
Orthorhombic, *P*2_1_2_1_2_1_	Mo *K*α radiation, λ = 0.71073 Å
Hall symbol: P 2ac 2ab	Cell parameters from 25 reflections
*a* = 6.1700 (12) Å	θ = 9–13°
*b* = 11.051 (2) Å	µ = 0.07 mm^−^^1^
*c* = 37.526 (8) Å	*T* = 293 K
*V* = 2558.7 (9) Å^3^	Block, white
*Z* = 4	0.30 × 0.20 × 0.10 mm

### Data collection

**Table:**

Enraf–Nonius CAD-4 diffractometer	2211 reflections with *I* > 2σ(*I*)
Radiation source: fine-focus sealed tube	*R*_int_ = 0.088
Graphite monochromator	θ_max_ = 25.4°, θ_min_ = 1.1°
ω/2θ scans	*h* = 0→7
Absorption correction: ψ scan (*CAD-4 Software*; North *et al.*, 1968)	*k* = 0→13
*T*_min_ = 0.978, *T*_max_ = 0.993	*l* = −45→45
5399 measured reflections	3 standard reflections every 200 reflections
4691 independent reflections	intensity decay: 1%

### Refinement

**Table:**

Refinement on *F*^2^	Primary atom site location: structure-invariant direct methods
Least-squares matrix: full	Secondary atom site location: difference Fourier map
*R*[*F*^2^ > 2σ(*F*^2^)] = 0.078	Hydrogen site location: inferred from neighbouring sites
*wR*(*F*^2^) = 0.183	H-atom parameters constrained
*S* = 1.00	*w* = 1/[σ^2^(*F*_o_^2^) + (0.060*P*)^2^] where *P* = (*F*_o_^2^ + 2*F*_c_^2^)/3
4691 reflections	(Δ/σ)_max_ < 0.001
298 parameters	Δρ_max_ = 0.16 e Å^−^^3^
1 restraint	Δρ_min_ = −0.17 e Å^−^^3^

### Special details

**Table:**

Geometry. All e.s.d.'s (except the e.s.d. in the dihedral angle between two l.s. planes) are estimated using the full covariance matrix. The cell e.s.d.'s are taken into account individually in the estimation of e.s.d.'s in distances, angles and torsion angles; correlations between e.s.d.'s in cell parameters are only used when they are defined by crystal symmetry. An approximate (isotropic) treatment of cell e.s.d.'s is used for estimating e.s.d.'s involving l.s. planes.
Refinement. Refinement of *F*^2^ against ALL reflections. The weighted *R*-factor *wR* and goodness of fit *S* are based on *F*^2^, conventional *R*-factors *R* are based on *F*, with *F* set to zero for negative *F*^2^. The threshold expression of *F*^2^ > σ(*F*^2^) is used only for calculating *R*-factors(gt) *etc*. and is not relevant to the choice of reflections for refinement. *R*-factors based on *F*^2^ are statistically about twice as large as those based on *F*, and *R*- factors based on ALL data will be even larger.

### Fractional atomic coordinates and isotropic or equivalent isotropic displacement parameters (Å^2^)

**Table:**

	*x*	*y*	*z*	*U*_iso_*/*U*_eq_	
N	0.1892 (7)	0.0800 (4)	0.03582 (11)	0.0798 (14)	
O1	0.1751 (9)	−0.3034 (4)	−0.08665 (11)	0.1262 (18)	
C1	0.3329 (16)	−0.3936 (6)	−0.0959 (2)	0.168 (4)	
H1A	0.2748	−0.4457	−0.1140	0.252*	
H1B	0.3680	−0.4407	−0.0751	0.252*	
H1C	0.4616	−0.3549	−0.1047	0.252*	
O2	0.2587 (6)	0.1742 (4)	0.06048 (9)	0.0806 (12)	
C2	0.2309 (11)	−0.2209 (5)	−0.06054 (14)	0.0738 (16)	
O3	−0.0806 (6)	0.1739 (4)	0.08121 (11)	0.1088 (16)	
C3	0.4223 (10)	−0.2218 (5)	−0.04202 (14)	0.0784 (17)	
H3A	0.5247	−0.2821	−0.0460	0.094*	
C4	0.4604 (9)	−0.1316 (5)	−0.01735 (13)	0.0712 (16)	
H4A	0.5918	−0.1306	−0.0052	0.085*	
C5	0.3105 (8)	−0.0432 (5)	−0.01018 (12)	0.0590 (13)	
C6	0.1146 (10)	−0.0433 (5)	−0.02848 (14)	0.0727 (16)	
H6A	0.0097	0.0150	−0.0238	0.087*	
C7	0.0789 (10)	−0.1324 (5)	−0.05395 (14)	0.0770 (16)	
H7A	−0.0501	−0.1323	−0.0668	0.092*	
C8	0.3520 (9)	0.0520 (6)	0.01648 (14)	0.0731 (16)	
C9	0.5748 (10)	0.1086 (7)	0.01919 (18)	0.135 (3)	
H9A	0.5759	0.1669	0.0381	0.202*	
H9B	0.6099	0.1479	−0.0029	0.202*	
H9C	0.6801	0.0468	0.0240	0.202*	
C10	0.1024 (8)	0.2116 (5)	0.08260 (13)	0.0616 (13)	
C11	0.1842 (8)	0.3063 (4)	0.10914 (12)	0.0548 (12)	
C12	0.1060 (9)	0.4277 (5)	0.09359 (13)	0.0724 (16)	
H12A	0.1925	0.4470	0.0728	0.087*	
H12B	−0.0433	0.4191	0.0859	0.087*	
C13	0.1212 (10)	0.5326 (4)	0.12026 (12)	0.0694 (15)	
H13A	0.2719	0.5473	0.1262	0.083*	
H13B	0.0627	0.6056	0.1096	0.083*	
C14	−0.0049 (9)	0.5020 (4)	0.15382 (12)	0.0615 (14)	
H14A	0.0084	0.5688	0.1704	0.074*	
H14B	−0.1570	0.4940	0.1477	0.074*	
C15	0.0682 (7)	0.3872 (4)	0.17213 (11)	0.0460 (11)	
C16	0.0614 (7)	0.2829 (4)	0.14438 (11)	0.0501 (11)	
H16A	−0.0913	0.2771	0.1374	0.060*	
C17	−0.0814 (7)	0.3510 (4)	0.20262 (12)	0.0525 (12)	
C18	−0.1403 (8)	0.2317 (5)	0.20981 (12)	0.0574 (13)	
C19	−0.0723 (9)	0.1300 (4)	0.18619 (13)	0.0711 (15)	
H19A	−0.1958	0.1046	0.1720	0.085*	
H19B	−0.0292	0.0620	0.2009	0.085*	
C20	0.1123 (8)	0.1615 (4)	0.16145 (13)	0.0659 (14)	
H20A	0.1276	0.0998	0.1432	0.079*	
H20B	0.2470	0.1664	0.1747	0.079*	
C21	−0.1556 (9)	0.4395 (5)	0.22626 (13)	0.0650 (14)	
H21A	−0.1182	0.5199	0.2222	0.078*	
C22	−0.2828 (9)	0.4119 (5)	0.25553 (13)	0.0658 (14)	
H22A	−0.3301	0.4733	0.2706	0.079*	
C23	−0.3401 (9)	0.2922 (5)	0.26245 (13)	0.0623 (13)	
C24	−0.2702 (9)	0.2063 (5)	0.23947 (13)	0.0696 (14)	
H24A	−0.3104	0.1264	0.2436	0.084*	
C25	0.4331 (8)	0.3015 (5)	0.11184 (15)	0.0794 (17)	
H25A	0.4764	0.2251	0.1217	0.119*	
H25B	0.4830	0.3658	0.1270	0.119*	
H25C	0.4950	0.3106	0.0885	0.119*	
C26	0.2970 (8)	0.4086 (4)	0.18935 (13)	0.0694 (15)	
H26A	0.2883	0.4739	0.2062	0.104*	
H26B	0.3996	0.4287	0.1710	0.104*	
H26C	0.3430	0.3363	0.2013	0.104*	
C27	−0.4765 (10)	0.2590 (5)	0.29540 (14)	0.0794 (17)	
H27A	−0.4753	0.1706	0.2971	0.095*	
C28	−0.3787 (12)	0.3065 (7)	0.32966 (14)	0.120 (3)	
H28A	−0.4703	0.2851	0.3494	0.181*	
H28B	−0.3660	0.3930	0.3283	0.181*	
H28C	−0.2377	0.2716	0.3330	0.181*	
C29	−0.7111 (11)	0.2963 (6)	0.29055 (18)	0.104 (2)	
H29A	−0.7919	0.2758	0.3116	0.157*	
H29B	−0.7712	0.2546	0.2704	0.157*	
H29C	−0.7187	0.3820	0.2866	0.157*	

### Atomic displacement parameters (Å^2^)

**Table:**

	*U*^11^	*U*^22^	*U*^33^	*U*^12^	*U*^13^	*U*^23^
N	0.063 (3)	0.111 (4)	0.065 (3)	−0.009 (3)	0.001 (3)	−0.033 (3)
O1	0.185 (5)	0.093 (3)	0.101 (3)	0.023 (4)	−0.032 (4)	−0.031 (3)
C1	0.250 (11)	0.095 (5)	0.158 (7)	0.084 (7)	−0.017 (8)	−0.045 (5)
O2	0.063 (2)	0.115 (3)	0.064 (2)	−0.027 (2)	0.018 (2)	−0.035 (2)
C2	0.092 (4)	0.070 (4)	0.059 (3)	0.011 (4)	−0.003 (3)	0.000 (3)
O3	0.054 (2)	0.151 (4)	0.121 (3)	−0.029 (3)	0.003 (3)	−0.074 (3)
C3	0.094 (5)	0.074 (4)	0.067 (3)	0.024 (4)	0.005 (4)	0.006 (3)
C4	0.059 (3)	0.091 (4)	0.063 (3)	0.025 (3)	−0.006 (3)	−0.003 (3)
C5	0.052 (3)	0.077 (4)	0.048 (3)	0.000 (3)	−0.001 (3)	−0.003 (2)
C6	0.077 (4)	0.075 (4)	0.066 (3)	0.009 (3)	0.000 (3)	−0.012 (3)
C7	0.075 (4)	0.081 (4)	0.075 (4)	0.016 (4)	−0.011 (3)	−0.010 (3)
C8	0.058 (3)	0.112 (5)	0.049 (3)	−0.001 (3)	−0.001 (3)	−0.011 (3)
C9	0.062 (4)	0.216 (9)	0.127 (6)	−0.019 (5)	0.012 (4)	−0.075 (6)
C10	0.052 (3)	0.071 (3)	0.062 (3)	−0.003 (3)	0.002 (3)	−0.015 (3)
C11	0.040 (2)	0.072 (3)	0.053 (3)	−0.009 (3)	0.001 (2)	−0.014 (3)
C12	0.072 (4)	0.088 (4)	0.058 (3)	−0.017 (3)	−0.004 (3)	−0.002 (3)
C13	0.089 (4)	0.055 (3)	0.065 (3)	−0.005 (3)	0.014 (3)	0.006 (3)
C14	0.069 (3)	0.049 (3)	0.067 (3)	−0.005 (3)	0.007 (3)	−0.001 (2)
C15	0.045 (3)	0.040 (2)	0.053 (3)	−0.005 (2)	−0.005 (2)	0.003 (2)
C16	0.037 (3)	0.053 (3)	0.060 (3)	0.000 (2)	−0.005 (2)	−0.004 (2)
C17	0.049 (3)	0.053 (3)	0.055 (3)	0.006 (3)	−0.012 (2)	0.011 (2)
C18	0.050 (3)	0.074 (3)	0.049 (3)	0.003 (3)	−0.003 (2)	0.011 (2)
C19	0.077 (4)	0.063 (3)	0.073 (4)	−0.005 (3)	−0.004 (3)	0.000 (3)
C20	0.067 (4)	0.053 (3)	0.077 (3)	0.010 (3)	−0.009 (3)	−0.004 (3)
C21	0.068 (3)	0.065 (3)	0.062 (3)	0.004 (3)	−0.003 (3)	−0.005 (3)
C22	0.068 (3)	0.076 (3)	0.054 (3)	0.011 (3)	−0.004 (3)	−0.008 (3)
C23	0.065 (3)	0.067 (3)	0.055 (3)	−0.003 (3)	−0.003 (3)	0.008 (3)
C24	0.071 (4)	0.067 (3)	0.070 (3)	0.003 (3)	−0.007 (3)	0.010 (3)
C25	0.058 (3)	0.098 (4)	0.083 (4)	−0.013 (4)	−0.005 (3)	−0.008 (3)
C26	0.057 (3)	0.085 (4)	0.066 (3)	−0.026 (3)	−0.016 (3)	−0.008 (3)
C27	0.073 (4)	0.101 (4)	0.064 (4)	0.012 (4)	0.019 (3)	0.021 (3)
C28	0.136 (6)	0.166 (7)	0.059 (4)	0.012 (6)	0.003 (4)	0.011 (4)
C29	0.092 (5)	0.110 (5)	0.111 (5)	0.013 (4)	0.022 (4)	0.014 (4)

### Geometric parameters (Å, º)

**Table:**

N—C8	1.277 (6)	C15—C17	1.523 (6)
N—O2	1.458 (5)	C15—C16	1.555 (5)
O1—C2	1.382 (6)	C15—C26	1.571 (6)
O1—C1	1.436 (7)	C16—C20	1.519 (6)
C1—H1A	0.9600	C16—H16A	0.9800
C1—H1B	0.9600	C17—C18	1.393 (6)
C1—H1C	0.9600	C17—C21	1.398 (6)
O2—C10	1.338 (5)	C18—C24	1.400 (6)
C2—C3	1.370 (8)	C18—C19	1.492 (6)
C2—C7	1.377 (7)	C19—C20	1.510 (6)
O3—C10	1.204 (5)	C19—H19A	0.9700
C3—C4	1.381 (7)	C19—H19B	0.9700
C3—H3A	0.9300	C20—H20A	0.9700
C4—C5	1.371 (6)	C20—H20B	0.9700
C4—H4A	0.9300	C21—C22	1.384 (6)
C5—C6	1.390 (7)	C21—H21A	0.9300
C5—C8	1.475 (7)	C22—C23	1.394 (6)
C6—C7	1.390 (7)	C22—H22A	0.9300
C6—H6A	0.9300	C23—C24	1.353 (6)
C7—H7A	0.9300	C23—C27	1.540 (7)
C8—C9	1.514 (8)	C24—H24A	0.9300
C9—H9A	0.9600	C25—H25A	0.9600
C9—H9B	0.9600	C25—H25B	0.9600
C9—H9C	0.9600	C25—H25C	0.9600
C10—C11	1.530 (6)	C26—H26A	0.9600
C11—C12	1.540 (6)	C26—H26B	0.9600
C11—C16	1.546 (6)	C26—H26C	0.9600
C11—C25	1.540 (6)	C27—C29	1.516 (8)
C12—C13	1.535 (6)	C27—C28	1.514 (8)
C12—H12A	0.9700	C27—H27A	0.9800
C12—H12B	0.9700	C28—H28A	0.9600
C13—C14	1.519 (6)	C28—H28B	0.9600
C13—H13A	0.9700	C28—H28C	0.9600
C13—H13B	0.9700	C29—H29A	0.9600
C14—C15	1.511 (6)	C29—H29B	0.9600
C14—H14A	0.9700	C29—H29C	0.9600
C14—H14B	0.9700		
			
C8—N—O2	107.6 (4)	C16—C15—C26	114.3 (4)
C2—O1—C1	117.4 (6)	C20—C16—C11	114.0 (4)
O1—C1—H1A	109.5	C20—C16—C15	111.5 (3)
O1—C1—H1B	109.5	C11—C16—C15	115.8 (4)
H1A—C1—H1B	109.5	C20—C16—H16A	104.7
O1—C1—H1C	109.5	C11—C16—H16A	104.7
H1A—C1—H1C	109.5	C15—C16—H16A	104.7
H1B—C1—H1C	109.5	C18—C17—C21	117.0 (5)
C10—O2—N	113.7 (4)	C18—C17—C15	123.5 (4)
C3—C2—C7	120.1 (5)	C21—C17—C15	119.4 (4)
C3—C2—O1	124.7 (6)	C17—C18—C24	119.6 (5)
C7—C2—O1	115.2 (6)	C17—C18—C19	121.6 (4)
C2—C3—C4	118.7 (5)	C24—C18—C19	118.8 (5)
C2—C3—H3A	120.6	C18—C19—C20	113.8 (4)
C4—C3—H3A	120.6	C18—C19—H19A	108.8
C5—C4—C3	122.1 (5)	C20—C19—H19A	108.8
C5—C4—H4A	119.0	C18—C19—H19B	108.8
C3—C4—H4A	119.0	C20—C19—H19B	108.8
C4—C5—C6	119.3 (5)	H19A—C19—H19B	107.7
C4—C5—C8	121.6 (5)	C19—C20—C16	107.9 (4)
C6—C5—C8	119.1 (5)	C19—C20—H20A	110.1
C7—C6—C5	118.5 (5)	C16—C20—H20A	110.1
C7—C6—H6A	120.7	C19—C20—H20B	110.1
C5—C6—H6A	120.7	C16—C20—H20B	110.1
C2—C7—C6	121.2 (5)	H20A—C20—H20B	108.4
C2—C7—H7A	119.4	C22—C21—C17	122.3 (5)
C6—C7—H7A	119.4	C22—C21—H21A	118.8
N—C8—C5	114.9 (5)	C17—C21—H21A	118.8
N—C8—C9	125.2 (5)	C23—C22—C21	120.1 (5)
C5—C8—C9	119.9 (5)	C23—C22—H22A	120.0
C8—C9—H9A	109.5	C21—C22—H22A	120.0
C8—C9—H9B	109.5	C24—C23—C22	117.8 (5)
H9A—C9—H9B	109.5	C24—C23—C27	121.3 (5)
C8—C9—H9C	109.5	C22—C23—C27	120.9 (5)
H9A—C9—H9C	109.5	C23—C24—C18	123.2 (5)
H9B—C9—H9C	109.5	C23—C24—H24A	118.4
O3—C10—O2	122.8 (5)	C18—C24—H24A	118.4
O3—C10—C11	125.0 (5)	C11—C25—H25A	109.5
O2—C10—C11	112.2 (4)	C11—C25—H25B	109.5
C10—C11—C12	104.2 (4)	H25A—C25—H25B	109.5
C10—C11—C16	106.3 (4)	C11—C25—H25C	109.5
C12—C11—C16	108.5 (4)	H25A—C25—H25C	109.5
C10—C11—C25	110.4 (4)	H25B—C25—H25C	109.5
C12—C11—C25	111.5 (4)	C15—C26—H26A	109.5
C16—C11—C25	115.3 (4)	C15—C26—H26B	109.5
C13—C12—C11	113.1 (4)	H26A—C26—H26B	109.5
C13—C12—H12A	109.0	C15—C26—H26C	109.5
C11—C12—H12A	109.0	H26A—C26—H26C	109.5
C13—C12—H12B	109.0	H26B—C26—H26C	109.5
C11—C12—H12B	109.0	C29—C27—C28	112.9 (6)
H12A—C12—H12B	107.8	C29—C27—C23	111.1 (5)
C14—C13—C12	110.0 (4)	C28—C27—C23	112.4 (5)
C14—C13—H13A	109.7	C29—C27—H27A	106.6
C12—C13—H13A	109.7	C28—C27—H27A	106.6
C14—C13—H13B	109.7	C23—C27—H27A	106.6
C12—C13—H13B	109.7	C27—C28—H28A	109.5
H13A—C13—H13B	108.2	C27—C28—H28B	109.5
C15—C14—C13	114.3 (4)	H28A—C28—H28B	109.5
C15—C14—H14A	108.7	C27—C28—H28C	109.5
C13—C14—H14A	108.7	H28A—C28—H28C	109.5
C15—C14—H14B	108.7	H28B—C28—H28C	109.5
C13—C14—H14B	108.7	C27—C29—H29A	109.5
H14A—C14—H14B	107.6	C27—C29—H29B	109.5
C14—C15—C17	112.4 (4)	H29A—C29—H29B	109.5
C14—C15—C16	108.0 (3)	C27—C29—H29C	109.5
C17—C15—C16	107.0 (3)	H29A—C29—H29C	109.5
C14—C15—C26	109.2 (4)	H29B—C29—H29C	109.5
C17—C15—C26	106.0 (4)		
			
C8—N—O2—C10	−179.7 (5)	C10—C11—C16—C15	−163.9 (4)
C1—O1—C2—C3	−2.5 (9)	C12—C11—C16—C15	−52.3 (5)
C1—O1—C2—C7	176.8 (6)	C25—C11—C16—C15	73.5 (5)
C7—C2—C3—C4	−1.2 (9)	C14—C15—C16—C20	−174.6 (4)
O1—C2—C3—C4	178.0 (5)	C17—C15—C16—C20	−53.4 (5)
C2—C3—C4—C5	1.8 (9)	C26—C15—C16—C20	63.6 (5)
C3—C4—C5—C6	−0.7 (8)	C14—C15—C16—C11	52.7 (5)
C3—C4—C5—C8	179.4 (5)	C17—C15—C16—C11	174.0 (4)
C4—C5—C6—C7	−1.0 (8)	C26—C15—C16—C11	−69.1 (5)
C8—C5—C6—C7	179.0 (5)	C14—C15—C17—C18	140.3 (4)
C3—C2—C7—C6	−0.4 (9)	C16—C15—C17—C18	21.9 (6)
O1—C2—C7—C6	−179.7 (5)	C26—C15—C17—C18	−100.4 (5)
C5—C6—C7—C2	1.5 (9)	C14—C15—C17—C21	−43.6 (6)
O2—N—C8—C5	179.8 (4)	C16—C15—C17—C21	−162.0 (4)
O2—N—C8—C9	−0.2 (9)	C26—C15—C17—C21	75.7 (5)
C4—C5—C8—N	−140.2 (6)	C21—C17—C18—C24	0.7 (6)
C6—C5—C8—N	39.9 (8)	C15—C17—C18—C24	176.9 (4)
C4—C5—C8—C9	39.9 (9)	C21—C17—C18—C19	179.3 (4)
C6—C5—C8—C9	−140.1 (6)	C15—C17—C18—C19	−4.4 (7)
N—O2—C10—O3	−2.7 (8)	C17—C18—C19—C20	16.9 (6)
N—O2—C10—C11	177.4 (4)	C24—C18—C19—C20	−164.4 (4)
O3—C10—C11—C12	−79.2 (7)	C18—C19—C20—C16	−46.7 (5)
O2—C10—C11—C12	100.7 (5)	C11—C16—C20—C19	−158.6 (4)
O3—C10—C11—C16	35.3 (8)	C15—C16—C20—C19	67.9 (5)
O2—C10—C11—C16	−144.8 (4)	C18—C17—C21—C22	−0.3 (7)
O3—C10—C11—C25	161.0 (6)	C15—C17—C21—C22	−176.7 (4)
O2—C10—C11—C25	−19.1 (6)	C17—C21—C22—C23	0.4 (8)
C10—C11—C12—C13	165.9 (4)	C21—C22—C23—C24	−1.0 (8)
C16—C11—C12—C13	53.0 (5)	C21—C22—C23—C27	178.4 (5)
C25—C11—C12—C13	−75.0 (6)	C22—C23—C24—C18	1.4 (8)
C11—C12—C13—C14	−56.2 (6)	C27—C23—C24—C18	−178.0 (5)
C12—C13—C14—C15	57.6 (6)	C17—C18—C24—C23	−1.3 (7)
C13—C14—C15—C17	−172.2 (4)	C19—C18—C24—C23	180.0 (5)
C13—C14—C15—C16	−54.5 (5)	C24—C23—C27—C29	−106.9 (6)
C13—C14—C15—C26	70.4 (5)	C22—C23—C27—C29	73.8 (7)
C10—C11—C16—C20	64.7 (5)	C24—C23—C27—C28	125.5 (6)
C12—C11—C16—C20	176.2 (4)	C22—C23—C27—C28	−53.8 (7)
C25—C11—C16—C20	−58.0 (6)		

## References

[bb1] Cui, Y. J., Rao, X. P., Shang, S. B., Song, J. & Gao, Y. Q. (2013). *Lett. Drug Des. Discov.* **10**, 102–110.

[bb2] Enraf–Nonius (1989). *CAD-4 Software* Enraf–Nonius, Delft, The Netherlands.

[bb3] Harms, K. & Wocadlo, S. (1995). *XCAD4* University of Marburg, Germany.

[bb4] Li, F., He, L., Song, Z. Q., Yao, J. C., Rao, X. P. & Li, H. T. (2008). *J. Pharm. Pharmacol.* **60**, 205–211. 10.1211/jpp.60.2.0009 18237468

[bb5] North, A. C. T., Phillips, D. C. & Mathews, F. S. (1968). *Acta Cryst.* A**24**, 351–359.

[bb6] Rao, X. P., Song, Z. Q., He, L. & Jia, W. H. (2008). *Chem. Pharm. Bull.* **56**,1575–1578. 10.1248/cpb.56.1575 18981608

[bb7] Rao, X.-P., Song, Z.-Q. & Shang, S.-B. (2009). *Acta Cryst.* E**65**, o2402. 10.1107/S1600536809035600 PMC2970241 21577865

[bb8] Sepulveda, B., Astudillo, L., Rodriguez, J., Yanez, T., Theoduloz, C. & Schmeda, G. (2005). *Pharm. Res.* **52**, 429–437. 10.1016/j.phrs.2005.06.004 16125407

[bb9] Sheldrick, G. M. (2008). *Acta Cryst.* A**64**, 112–122. 10.1107/S0108767307043930 18156677

