# Supplementary figures and images for: Crystal structure of (E)-1-(4-meth­oxy­phen­yl)ethanone O-de­hydro­abietyloxime
Source: Acta Crystallogr Sect E Struct Rep Online. 2014 Aug 1;70(Pt 9):o948. doi: 10.1107/S1600536814016882 (PMC4186138; doi:10.1107/S1600536814016882)

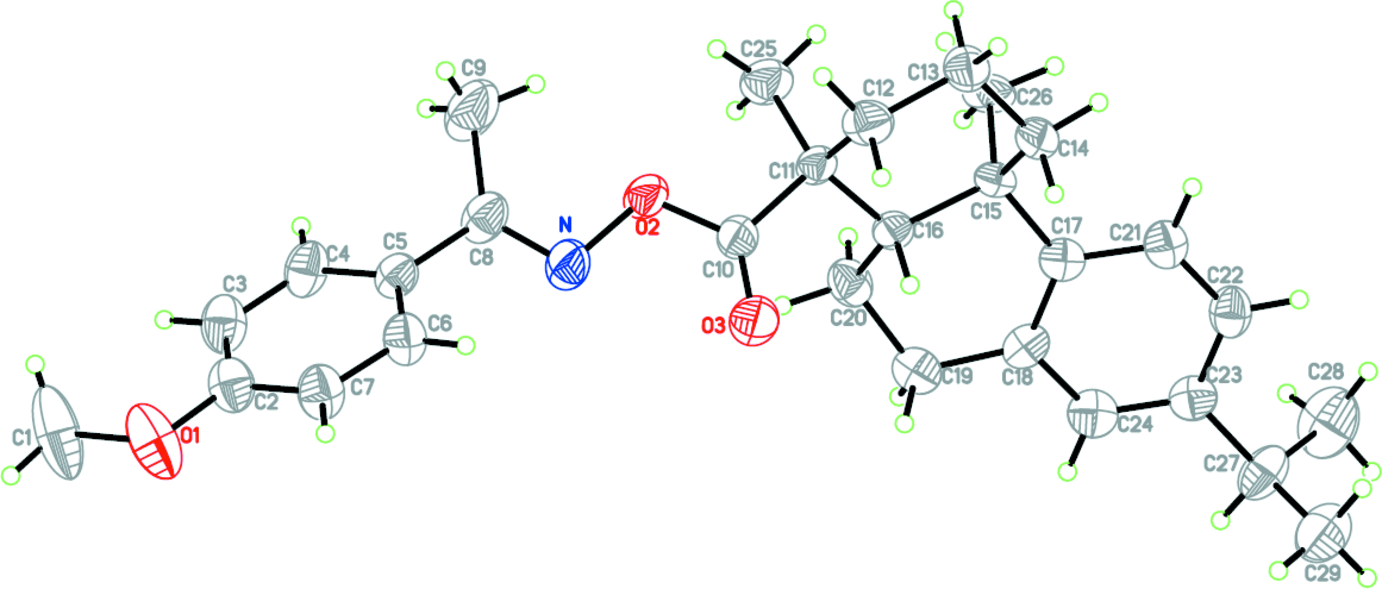

Supplement: Supplementary file 3 [file e-70-0o948-fig1.tif]
